# Modelling hormonal response and development^☆^

**DOI:** 10.1016/j.tplants.2014.02.004

**Published:** 2014-05

**Authors:** Ute Voß, Anthony Bishopp, Etienne Farcot, Malcolm J. Bennett

**Affiliations:** 1 Centre for Plant Integrative Biology, University of Nottingham, Nottingham, LE12 5RD, UK; 2 School of Mathematical Sciences, University of Nottingham, Nottingham, NG7 2RD, UK

**Keywords:** modelling, hormone signalling, systems biology, multiscale modelling

## Abstract

As our knowledge of the complexity of hormone homeostasis, transport, perception, and response increases, and their outputs become less intuitive, modelling is set to become more important. Initial modelling efforts have focused on hormone transport and response pathways. However, we now need to move beyond the network scales and use multicellular and multiscale modelling approaches to predict emergent properties at different scales. Here we review some examples where such approaches have been successful, for example, auxin–cytokinin crosstalk regulating root vascular development or a study of lateral root emergence where an iterative cycle of modelling and experiments lead to the identification of an overlooked role for PIN3. Finally, we discuss some of the remaining biological and technical challenges.

## Highlights

• Modelling has helped to understand the complex network structure of single hormonal pathways, but has also provided insights into hormone activity at many levels.
• We describe how multiple hormones have been incorporated into new models.
• We explore future challenges in integrating different models.
• We propose that future models need to be more realistic by capturing more geometrical, mechanical as well as biological data.

## Understanding nonlinear networks

As our knowledge of hormone-regulated plant development increases, researchers are realising that the underlying components (e.g., proteins, cells, and organs) are embedded within or contain highly complex networks (reviewed in [1]). Although comparing different mutants and their phenotypes in model systems such as *Arabidopsis* (*Arabidopsis thaliana*) has worked well to unravel linear hormone response pathways, this is insufficient for understanding the dynamics of nonlinear networks (reviewed in [2,3]). As a consequence, there is an increasing interest in moving away from studying individual gene products and simple linear pathways, and instead dissecting more complex relationships between multiple components within nonlinear pathways [4]. For example, the majority of hormone transduction pathways include several negative or positive feedback loops, or feed-forward loops, each of which can have different functions, such as attenuating or amplifying outputs. The intertwinement of these loops makes the output difficult to predict (i.e., nonintuitive) using logic alone. Systems approaches involving mathematical or computational models are enabling researchers to simulate the behaviours of these nonlinear networks and predict emergent properties (reviewed in [5]).

In this review, we discuss how modelling approaches help researchers understand the mechanistic behaviour of hormones and how they control organ growth and development. We describe models capturing hormone transport and response pathways, and discuss increasingly complex models that integrate multiple hormone response pathways, tissues, and/or scales. A more general overview of the principals of systems modelling can be found in [3–6].

## Hormone transport models

**Figure 1:**
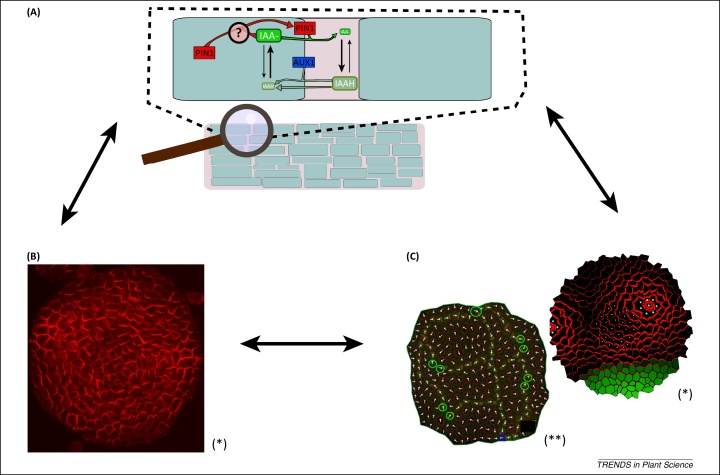
Models of auxin transport in the shoot apex. **(A)** At the cell level, auxin is actively transported by PIN (efflux) and AUX (influx) proteins, in addition to the natural influx of protonated auxin. Abbreviation: IAAH, indole-3-acetamide hydrolase. The polar localisation of PIN on the membranes is believed to be auxin-dependent, but the exact mechanism is unknown, as indicated by the question mark. Black arrows represent chemical reactions (thickness indicates relative rates). Coloured arrows represent transport of the substance bearing the same colour. **(B)** Experimental data, which often consists of microscope images of fluorescent reporters for auxin response and/or antibody-based localisation of PIN subcellular distribution (in red), are incorporated into multicellular computational models **(C)** to make predictions about auxin distribution patterns (denoted in green). In (C) left, white arrows represent PIN polarity, green circles are auxin sources, and blue triangles auxin sinks. In (C) right, white dots mark cells from the central zone. (B) and (C) are reproduced, with permission, from [25], indicated by (*) and [27], indicated by (**).

Models have been used to study the intercellular transport of several hormones, including cytokinin (e.g., [7]), auxin (e.g., [8]), and gibberellin (GA; e.g., [9]). This cell to cell transport involves export across the plasma membrane, diffusion through the apoplast, and import into neighbouring cells via the plasma membrane. Membrane transport can be either active or passive. Unfortunately, apoplastic transport is often neglected in hormone transport models, despite the availability of quantitative data describing this process [10]. However, because transporters for GA and cytokinin have not been characterised, these hormones are frequently treated as diffusing passively between cells. Auxin is actively transported in a polar manner by several classes of specialised proteins, including: AUX1/LAX proteins that mediate auxin influx together with membrane diffusion of protonated auxin, and the PIN-FORMED (PIN) family members that function as auxin efflux carriers and exhibit polarised localisation on specific cell faces [11,12]. The polar distribution of PIN proteins is believed to be auxin-dependent [13], although the precise underlying mechanism is unknown. Transport of auxin from cell to cell results in localised asymmetries in the distribution of auxin and these drive various developmental processes. Auxin transport occurs on multiple scales and incorporates the subcellular redistribution of PIN transporters, long-range transport of auxin across the whole plant, as well as tight control of auxin perception at the organ and tissue scales. This complexity has fostered many mathematical and computational models that are helping to explain and subsequently predict experimental data (see Figure 1 for an illustration of some key aspects; [14]).

Early auxin transport models formalised the ideas proposed by Tsvi Sachs in the late 1960s to explain vein patterns [15]. These flux-based models hypothesised that the flux of auxin through cell membranes reinforces itself [16–18], resulting in sharply defined asymmetries in auxin concentration. As PIN proteins were unknown at this time, auxin transport was described using abstract variables representing the permeability of cell membranes and whose value was an increasing function of the directed auxin flux. This model confirmed the plausibility of Sachs’ hypothesis by producing realistic vein patterns.

Today technology has enabled more advanced simulations, and computational models can test different hypotheses for auxin transport. Some studies relied on experimentally determined patterns of PIN localisation, generated by confocal microscopes and computer-based image analysis, to run simulations including auxin as the only dynamic variable. These simulations confirmed that PIN transporters are sufficient to describe auxin patterns in the shoot [19] and root [8]. Computer simulations have also revealed the contribution of auxin influx proteins (such as AUX1) at the root apex [20].

In parallel to these studies where PIN polarity was set up based on experimental data, several models were developed in which the PIN distribution dynamically changed as a function of auxin. As an alternative to flux-based models, so-called gradient-based models were introduced, where local accumulation of transporters depends on local differences in auxin concentration and follows increasing gradient directions, rather than the flux across cell membranes [21,22]. Such models can account for the distribution of auxin maxima in the shoot apical meristem (SAM); local maxima of auxin appear at the loci of emerging primordia, surrounded by local inhibition fields (through auxin depletion), which induce the characteristic, very regular patterns of leaf phyllotaxy. Shortly after, a new variant of the flux-based models was developed in which permeability was interpreted in terms of local PIN concentration. This model was able to reproduce plausible vein patterns [23,24], as well as simulating phyllotactic patterns in the SAM [25]. These studies drove further research and generated controversy over the question of whether PIN accumulation was regulated by auxin flux, its gradient, or some other mechanisms that rely on neither fluxes nor gradients of auxin. These include a regulation of cell polarity by mechanical stress [26], as well as a scenario where intracellular PIN distribution is jointly regulated by a hypothetical extracellular auxin receptor and intracellular auxin signalling [27].

Consequently, several models relying on various sets of hypotheses were proposed and analysed by different groups (reviewed in [28]). Such studies include a composite model, incorporating both flux-based and gradient-based PIN regulation in a tissue-dependent way [29]. Other theoretical studies considered the general patterning abilities of different models. This includes abstract models discussing the auto-organisation of independently polarised cells through spatial coupling [30]. For instance, in a similar way to Alan Turing's reaction diffusion models, stripes or spot-like patterns have been generated using both gradient-based models [31] and flux-based models [32]. Using tools from nonlinear dynamic systems theory, behaviours, including more complex than fixed patterns of auxin, were observed; for both gradient- [33] and flux-based [34] models, this includes travelling waves and stable oscillations of auxin in a tissue. Remarkably, auxin-regulated genes have been observed experimentally to oscillate in roots [35,36]. Mathematical models also suggest the possibility of oscillations of the auxin signalling pathway [37], at the single cell level. This raises the possibility of a situation where travelling waves of auxin are periodically transported along the root, whereas auxin responsive components oscillate in individual cells, leading to potentially complex temporal patterns of expression. This complexity might be compatible with oscillations of auxin, despite the data in [36], which shows that the levels of the indole-3-acetic acid protein IAA19, a direct auxin target, do not oscillate.

## Modelling hormone signalling networks

Translating spatiotemporal variations in hormone levels into differences in cellular behaviours requires models that capture regulatory networks incorporating gene transcription, translation, and protein–protein interactions (e.g., [38]). Significant progress has been made in developing mathematical models of plant hormone response pathways with increasingly complex network dynamics. Gene regulatory network models have been developed for several hormones, including abscisic acid (ABA) [39], GA [40], cytokinin [38], and auxin [37], using different types of modelling approaches, including Boolean, stochastic, and ordinary differential equation (ODE) models (see Glossary for these modelling terms; reviewed in [4,5]).

**Figure 2:**
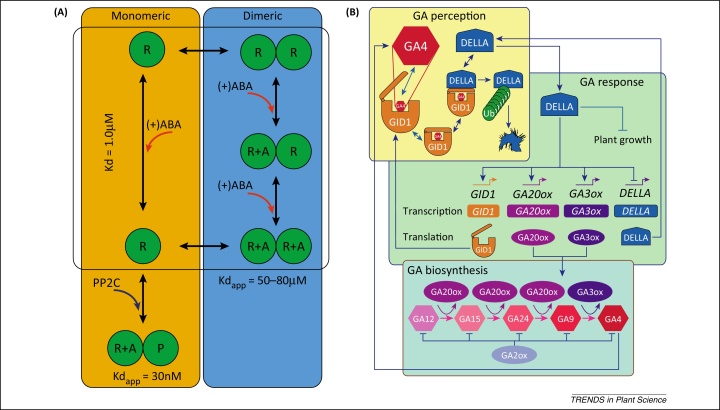
Models of the abscisic acid (ABA) and the gibberellin (GA) hormone signalling network. **(A)** Model of ABA receptor activation showing the formation of receptor–ABA–PP2C (R–A–P) ternary complexes for the monomeric and the dimeric PYR/PYL/RCAR proteins considered in the modelling study [52]. The dissociation constants (*K*_d_) for the reactions were measured experimentally and used to parameterise the model. Adapted from [39]. **(B)** The three functional modules of the GA signalling network (perception, response, and biosynthesis) are shown. Perception (yellow box): GA4 first binds to the GID receptor and the complex then interacts with DELLA proteins, leading to the ubiquitination (denoted with a green chain) and degradation of DELLA proteins. Response (green box): *GID1*, *GA20OX*, and *GA3OX* genes are transcriptionally activated by DELLA proteins but repress their own transcription. Biosynthesis (blue box): GA12 is converted to GA15 then to GA24, and finally to GA9 by the GA20ox enzyme. GA9 is then converted to GA4 by the GA3ox enzyme. Hence, DELLA-mediated upregulation of GA biosynthesis transiently elevates the levels of the hormone and the GID1 receptor, leading to DELLA degradation, thus creating a negative feedback loop. Reproduced, with permission, from [40].

A stochastic model has been developed to study ABA signal perception [39]. ABA is a key hormone regulating root growth and responses to biotic and abiotic stresses. ABA binds to a family of intracellular receptors termed pyrabactin resistance (PYR), PYR1-like (PYL) or regulatory component of abscisic acid receptor (RCAR) proteins (Figure 2A). ABA binding promotes the formation of complexes involving the PYR, PYL, and RCAR receptors and several types of protein phosphatase type 2C (PP2C) proteins that activate ABA responses. PYR, PYL, and RCAR family members either bind ABA as a monomer or as a dimer. This network of interactions was captured in a model that was used to probe ABA response when both monomeric and dimeric PYR, PYL, and RCAR receptors compete for ABA and PP2C molecules [39]. The model predicted that monomeric receptors have a competitive advantage for binding, particularly at lower ABA concentrations. Hence, the receptor composition of a given tissue and their oligomerisation properties will impact ABA responsiveness [39].

Arguably, the most complex model developed to date for a hormone network simultaneously captures the perception, response, and biosynthesis pathways for GA [40]. GA is crucial for seed germination, promoting growth and floral development. GA binds the GIBBERELLIN-INSENSITIVE DWARF 1 (GID1) receptor and this induces GID1, DELLA and the F-box protein SLEEPY1 (SLY1)/GID2 to interact, leading to DELLA ubiquitination and degradation (Figure 2B). DELLA degradation releases the transcription factors PHYTOCHROME-INTERACTING FACTOR 3 (PIF3) and PIF4 and activates expression of GA-responsive genes [41,42]. Mathematical modelling of the GA perception machinery has predicted that conformational changes in the GA receptor control the time scale of the response. This model also predicted the importance of feedback loops on several levels of the network and how these loops interact to generate the signalling outputs that had previously been observed experimentally. This model captured not only downstream signalling events but also the biosynthesis of GA, but was able to reproduce quantitative biological data precisely [40].

## Increasing complexity as multiple hormone response pathways interact

Many hormone response pathways interact through shared components [43]. For instance, GA [44] and cytokinin [45] regulate auxin efflux carrier abundance. Similarly, cytokinin promotes the transcription of Aux/IAAs and, thus, reduces PIN expression [46], whereas auxin promotes the transcription of certain cytokinin signalling repressors in a tissue-specific context [47,48]. Given the complexity of these interactions, mathematical models have an essential role in understanding the effects of perturbing these networks and determining how multiple signals integrate to control development and growth.

The first model to consider hormone signal integration investigated the interaction between auxin and brassinosteroids (BRs) during shoot vascular patterning [49]. The shoot vascular tissues contain alternating bundles of phloem and xylem arranged around the perimeter of the vascular cylinder, and the position of these bundles coincides with localised peaks in expression of the auxin sensor DR5 [49]. A mathematical model was generated to simulate auxin movement in a ring of cells and it was found that an appropriate asymmetric localisation of efflux proteins was able to recreate a similar pattern of peaks in auxin as observed with the DR5 reporter [49]. BR-related mutants alter both the number of bundles and the size of the shoot vascular cylinder [50]. This effect was taken into account by altering the size of the ring of cells and this increased the number of auxin peaks [49], providing a framework for the coordinated control of shoot vascular patterning with BR indirectly regulating auxin signalling through changes in tissue geometry.

Further studies have investigated the interaction between BRs and auxin at a molecular level. Based on a Boolean logic-based approach, a model of the core auxin signalling and transport machinery, as well as BR signalling and biosynthesis machinery was created [51]. When either of these networks was supplied with an initial input they reached a quasi-steady state, including an oscillating developmental output. To integrate these models, the auxin and BR-responsive output was linked to a common developmental output representing the coregulation of cell elongation [52]. In addition, direct interactions were included where BIN2 can phosphorylate the AUXIN RESPONSE FACTOR 2 (ARF2) and inhibit its activity [53], and by introducing the auxin-mediated activation of BREVIS RADIX (BRX), through transcription or via promoting transfer of BRX to the nucleus where it presumably regulates transcription with NGATHA (NGA) transcription factors [54,55]. BRX is likely to impinge on BR biosynthesis [54,55] and this was also included in the model. To account for unknowns in the experimental data, the authors compared the topology of different variants of this model, including BRX activating or repressing NGA, BRX activating ARF2 or not, as well as combinations thereof. Using these topologies in simulations representing wild type or mutants, where either BRX or ARF2 were absent, enabled them to propose a most parsimonious model incorporating the current experimental data as well as predicting a new role of BRX in inhibiting auxin biosynthesis [51].

The gene regulatory network underlying the patterning of the SAM [6] has also been the focus for several modelling papers investigating the regulatory interaction between the peptide CLAVATA3 (CLV3) and the transcription factor WUSCHEL (WUS) [56–59]. The development of a sophisticated model incorporating a negative feedback among cytokinin signalling, biosynthesis, and WUS was able to generate a mechanism for correctly positioning the domain of WUS expression in the SAM. This has subsequently been backed up with experimental data, where the spatial pattern of cytokinin response and biosynthesis genes in wild type and *clv3* meristems were investigated [60].

Mathematical models provide a means to understand complex, nonintuitive interactions. For example, a complex interaction has been identified by studying the interaction of the transcription factor *PHABULOSA* (*PHB*) and cytokinin in controlling root meristem size, where cytokinin signalling regulates microRNA165/166. Both cytokinin and microRNA165/166 jointly regulate *PHB*, with PHB also promoting cytokinin biosynthesis [61]. It was initially unclear what function such a complicated regulatory network would provide. However, by generating a one-dimensional model of this system and running simulations of this molecular network with, and without, the regulation of microRNA165/166 by cytokinin, it was discovered that this regulatory loop dampens the reduction and accelerates the recovery of PHB levels as cytokinin levels fluctuate [61].

Ethylene is a gaseous plant hormone that is involved in regulating seed germination, cell elongation, fruit ripening, as well as organ senescence and abscission (reviewed in [62]). Ethylene signals through its receptor ETHYLENE RESPONSE FACTOR1 (ERF1) that activates the expression of downstream genes via a MITOGEN ACTIVATED PROTEIN KINASE (MAPK) module. Ethylene signalling activates genes such as *PLANT DEFENSIN1 (PDF1)*, which is also induced by jasmonic acid (JA) [63], or inhibits gene expression, for example, of the auxin-regulated *ARF2* [64]. A continuous model was developed to describe the activation dynamics of *ERF1* with different ethylene concentrations and compare dose–response curves form the model as well as from experimental data. The ability of the model to reproduce biological data suggests that all key components of this pathway have been incorporated in the model. In addition, the model predicts that the changes in dose–response could be, at least partially, due to changes in ERF1 at different ethylene concentrations, in particular by filtering stochastic and rapid chaotic fluctuations in ethylene levels [65].

Examples of hormonal crosstalk between three hormonal pathways exist, because auxin, ethylene, and cytokinin are connected by the peptide POLARIS (PLS). PLS dampens the ethylene and cytokinin response and positively regulates auxin homeostasis and transport [66,67]. A model for PLS-mediated crosstalk could reproduce quantitative experimental data with available mutants. Based on this model, the design of new experiments provided novel insights into PLS function: PLS quantitatively regulates auxin biosynthesis and transport, and thereby controls auxin concentration in a particular cell. This model could also predict a more precise mechanism for the interaction between PLS and the ethylene receptor, based on the reproduction of quantitative biological data [68].

## Modelling hormonal response at a multicellular level

Communication between cells is essential to ensure that their growth and development are coordinated to pattern a tissue. In plants, such communication often involves hormones, moving between adjacent cells and interacting with their signalling networks. In many cases, the dynamics depend on complex regulation at cellular and subcellular scales. Understanding how this regulation produces the dynamic tissue scale distribution is often nonintuitive, making multicellular modelling based on realistic tissue geometries an essential part of the research process.

**Figure 3:**
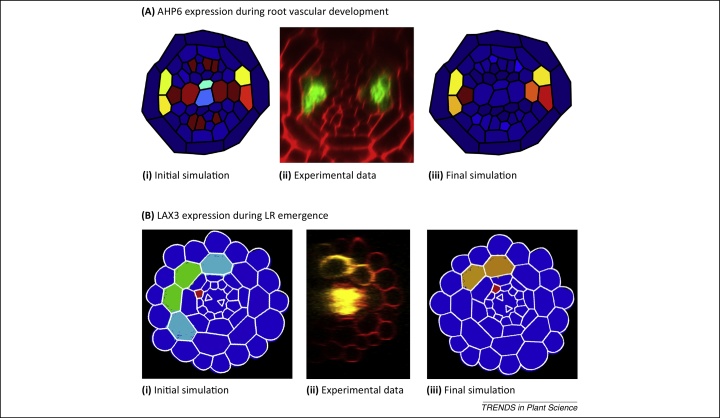
Multiscale models of hormone-regulated root development. **(A)** Initial models predicted that the current interactions between the known components regulating root vascular patterns were insufficient to correctly predict the expression of AHP6 and other key components in a multiscale model (**i**) with PIN proteins localised as they have been experimentally observed **(ii)**. However, when an additional inhibitor of cytokinin and the catalytic degradation of microRNA were incorporated into this model, it was able to predict AHP6 response patterns closely resembling those observed experimentally **(iii)**. **(B)** LAX3 expression is restricted to two cells overlying the LRP **(ii)**. Initial attempts to model this, using three-dimensional cell and tissue geometries, were unable to robustly restrict LAX3 expression to two cells **(i)**. However, by including an auxin efflux carrier into the model (subsequently identified as PIN3) and controlling the order of activation, the model was able to restrict LAX3 activity to just two cortical cells **(iii)**. In both sets of images, the expression of AHP6 or LAX3 is shown as a heat map, with red representing the highest expression. Images reproduced, with permission, from [69] and [73].

A two-dimensional multicellular model has recently been developed to probe how the interactions among auxin, cytokinin, and microRNA signals determine root vascular patterning [69]. This model included previously published findings of a mutually inhibitory interaction between auxin and cytokinin, whereby the auxin response activates the expression of the cytokinin signalling inhibitor *ARABIDOPSIS HISTIDINE PHOSPHOTRANSFER PROTEIN 6* (*AHP6*), the cytokinin response regulates the PIN class of auxin efflux carriers [48], and SHORT ROOT (SHR) promotes the expression of mobile *microRNA165/166*; this degrades *PHB* messenger RNA (mRNA) to form a gradient of *PHB* mRNA that controls the specification of xylem and inhibits *AHP6* expression [70,71]. The authors parameterised these components and incorporated them within a multicellular template representing the root vascular tissues and localised the PINs as they had been experimentally observed. They found that the published gene regulatory networks were insufficient to maintain stable expression patterns of key marker genes as observed experimentally (Figure 3A, panels i, ii). When the authors incorporated an additional regulator of cytokinin and introduced the catalytic degradation of both microRNA and *PHB* mRNA on binding, they were able to reproduce the experimentally observed patterns of all key markers within a flat field of both auxin and cytokinin (Figure 3A, panel iii).

A three-dimensional multicellular model was recently developed to study the striking expression pattern of the auxin influx carrier LAX3 during lateral root emergence. In *Arabidopsis*, lateral roots originate from pericycle cells deep within the primary root. A previous modelling study has shown that changes in cell shape caused by bending coupled with auxin-induced AUX1 expression can cause auxin maxima to form at bending sites corresponding with the growth of lateral roots [72]. New lateral root primordia (LRP) have to emerge through several overlaying tissues (Figure 3B, panel i). Auxin produced in new LRP is transported towards the outer tissues where it triggers cell separation by inducing both the auxin influx carrier LAX3 and cell wall remodelling enzymes. *LAX3* is expressed in just two cell files overlaying new LRP (Figure 3B, panel ii). To understand how *LAX3* spatial expression is regulated, a multicellular model was developed that captures the network regulating its expression and auxin transport within realistic three-dimensional cell and tissue geometries. Despite detailed knowledge about the regulatory components that control auxin-inducible *LAX3* expression in cortical cells overlaying new LRP, the molecular and tissue scale mechanisms controlling its highly specific expression pattern were unclear. In this study, the authors initially demonstrated that new LRP are able to channel auxin to overlaying cortical cells and induce *LAX3* expression. They then developed a mathematical model of the regulatory network controlling *LAX3* induction and coupled it to one for auxin movement in a realistic three-dimensional multicellular geometry (Figure 3B, panel iii). The model enabled the mechanisms regulating the spatial expression pattern of the influx carrier to be unravelled [73]. In particular, an iterative cycle of modelling and experimental perturbations suggested the existence of a new regulatory component, the auxin efflux carrier PIN3. By testing how robust the model is to natural variations in tissue geometry and auxin source, the authors concluded that PIN3 plays a key role. In addition, the model predicted that the *LAX3* expression pattern requires the sequential induction of auxin efflux and influx carriers (Figure 3B, panel iii), which was later experimentally demonstrated to be the case (Figure 3B, panel ii). Hence, the multicellular model predicted that the localisation of the auxin source, together with sequential induction of *PIN3* and *LAX3*, led to sharp *LAX3* expression patterns that are robust to variations in both the tissue geometry and the magnitude of the auxin source.

## Multiscale modelling of hormone signalling

When studying hormone-regulated growth and development, it is sometimes necessary to capture biological processes occurring at more than one physical and/or temporal scale. Multiscale models consider behaviours on two or more physical scales or at different temporal scales, ranging from minutes for transcription to weeks for developmental adaptation. To date, only a few multiscale models have been developed to address plant developmental questions. We have selected a multiscale model of GA dynamics in the *Arabidopsis* root elongation zone to illustrate the potential of developing multiscale models to probe the mechanisms underlying complex, nonlinear hormonal-regulated biological processes [9]. The authors determined cell growth (using experimental measurements) and simulated GA dilution, diffusion of GA between cellular compartments and the response network through which GA degrades the growth-repressing DELLA proteins (using ODEs). The model suggested that as cells pass through the elongation zone, dilution (rather than degradation) creates a declining GA concentration, leading to spatial gradients of *DELLA* mRNA and protein abundance. This model prediction was confirmed experimentally using knockout mutants lacking every root-expressed GA 2-oxidase degradation enzyme. The study also considered the dynamics in plants treated with paclobutrazol (an inhibitor of GA biosynthesis) and plants with mutations in the GA biosynthesis and signalling pathways, suggesting that the growth rates appear to reflect the fold change in DELLA as cells traverse the elongation zone. Furthermore, the model provided new insights into the previously confusing phenotype exhibited in the *ga1-3 gai-t6 rga-24* triple mutant. The model demonstrated that the effect of the *ga1–3* mutation in reducing GA biosynthesis (leading to higher levels of functional DELLA) counteracted the effect of the *gai-t6 rga-24* double mutation in reducing the translation of functional DELLA; if these two processes are suitably balanced, the levels of functional DELLA are similar to wild type, explaining why the triple mutant exhibits normal cell elongation. In summary, by assimilating a range of data and knowledge, the model predicted the dominant effect of GA dilution (rather than degradation) on the emergent DELLA distribution, providing new insights into the growth regulation of GA. This example suggests the utility of, and challenges faced by, adopting a multiscale modelling approach.

## Future challenges

This review highlights how mathematical and computational models are proving invaluable tools with which to generate new mechanistic insights into hormone-regulated plant growth and development. Although great progress has been made, several major issues remain to be addressed in order to develop models that better reflect biological reality: these include capturing enough biological information in the model, in particular quantitative data, integrating mechanics, integrating models, and the development of digital models.

### Capturing enough biological information in the model

Although the adage ‘as simple as possible but no simpler’ applies, such that models are of necessity abstractions of biological reality and seek to reflect and, hence, enhance the understanding of the key hormone processes of interest without obscuring these processes by including illusory levels of complexity. Nevertheless, models frequently omit crucial processes such as biosynthesis and degradation that can play key roles, together with transport, in determining the cellular abundance of hormones. By integrating these processes into models, unexpected mechanistic insights often emerge. This was predicted by modelling GA: it was discovered that dilution rather than degradation caused the declining GA concentrations seen in the elongation zone [9]. Capturing realistic cell and tissue geometries is often necessary to fully appreciate the importance of the hormone response network composition and organisation. This was recently suggested in a model of lateral root emergence when testing how robust a multicellular model was to natural variations in tissue geometry suggested a hitherto overlooked role for PIN3 [73]. Similarly, the functional importance of temporal regulation during hormone responses has received limited discussion in the plant field but is evidently crucial to ensure a robust output from signalling pathways. For example, studying the importance of dynamics in the lateral root emergence network led to the discovery that the efflux carrier *PIN3* and then the influx carrier *LAX3* must be induced sequentially [73].

### Generating more quantitative data and models

Only realistic models can make realistic biological predictions that can subsequently be confirmed by experiments. To improve models more quantitative data need to be generated and incorporated in models. This requires biologists to change the way they design, perform, and analyse experiments, and subsequently modellers to use data available in the literature more extensively. Better communication between both fields would help this problem and more importantly allow iterative cycles of model perturbation and experimental validation, as this is the real strength of systems biology.

### Integrating mechanics

To simulate growth, researchers must integrate models of mechanics with regulatory networks and/or cell and tissue geometries. Given that mechanical properties of plant cells are dominated by their walls, vertex-based models provide a natural framework for computational simulation of tissue scale mechanics [22,74–76]. More detailed cell based mechanical models in the SAM have used a finite element mesh that contains every cell wall of the tissue [76]. At the tissue scale, the organ can be treated as a continuum: typically, these recapitulate the viscoplastic behaviour embodied in the Lockhart equation, but are then amenable to traditional engineering analysis, for example, using finite element methods. In both vertex-based and continuum approaches, the important contribution of multiscale methods is to relate tissue level properties systematically to processes occurring at the level of the cell wall, and to enable mechanics to be coupled to the biological processes driving organ growth and development.

### Integrating models

This review has highlighted many models that would greatly benefit researchers if they could be assembled into unified frameworks. Nevertheless, model integration is challenging for several reasons. Firstly, models often operate at distinct temporal scales. For example, mechanical processes are often assumed to be much faster than biochemical processes. Secondly, models often operate at different spatial scales. For example, coupling a two-dimensional mechanical model of root tissues at a subcellular resolution with a two-dimensional model of auxin transport designed at cellular resolution would require that cell geometries are updated throughout time by the mechanical model. Thirdly, combining different types of mathematical models, for example, Boolean, stochastic, ODE, and partial differential equation (PDE), can often prove challenging [77]. Creating combinations of models often represents a large amount of theoretical and experimental effort. Hence, it is important that these are freely available to the community as shared datasets, open-source model formats, such as systems biology mark-up language (SBML [78]) and CellML [79], or common modelling software platforms, such as VV [80], OpenAlea [81], or MorphoGraphX [82]. Finally, most models of hormonal response simplify the network of hormone response components to consider only one of each type of response regulator. However, we are becoming increasingly aware that specificity between individual signalling components can mediate a different developmental response. As we try to integrate models of different processes occurring within organs such as the root, we will have to introduce multiple response regulators to allow a single hormone to signal multiple independent responses.

### Towards digital plant models

To date, models of hormone-regulated plant growth and development have focused on individual organs or parts of organs for obvious reasons. However, developing a mechanistic model of a whole plant represents a logical next step. To date, models of diverse root system subprocesses have been developed at different scales. Compared with the initial approaches in systems biology, most of these models make explicit use of spatial information. Such spatial information represents different aspects of realistic root structures and can take the form of a continuous medium, a branching structure of connected elements (e.g., root meristems), a multicellular population, or a set of interacting subcellular compartments. By progressively integrating more functional aspects into these realistic representations, researchers are creating a new generation of models [83,84]. Functional structural plant models (FSPMs) provide a promising platform with which to create a digital plant model. Compared with many previous FSPMs developed on aerial parts (e.g., [85]) and on root systems [86,87], recent FSPMs also integrate gene regulation and signalling as a new dimension in the analysis of development [88,89]. Through the combined modelling of genetic networks, physiological processes and spatial interaction between components, a new generation of FSPMs is being developed that opens the way to building digital versions of real plants [90,91] and testing biological hypotheses *in silico* [25,92].

## Glossary

Boolean: refers to a class of models where variables take only two possible values, traditionally denoted 0 and 1 as this allows to reduce a number of analyses to a calculation in a so-called Boolean algebra. A typical example consists of representing gene transcription as being on-going (value 1) or not (value 0).
Continuum approach: refers to a class of models where variables are represented by real numbers and can thus vary infinitesimally. Such models traditionally take the form of ordinary or partial differential equations (abbreviated ODE or PDE, respectively), depending on whether there is a continuous spatial domain (PDE) or not (ODE).
Finite element method: a numerical method that allows the approximate calculation of solutions of PDEs. The core principle is to discretise the continuous spatial domain using triangulation, and to locally approximate the PDE by a simpler problem on each triangle.
Lockhart equation: a particular differential equation that is often used to represent mechanics of a growing tissue.
Multiscale modelling: models which consider important features at multiple scales, particularly multiple spatial and (or) temporal scales.
Network scale modelling: models which consider important features at the gene or protein network scale.
Ordinary differential equation (ODE) model: a model relying on ordinary differential equations.
Stochastic: refers to a class of models where variables are represented by probabilities, and evolution involves some randomness.
Vertex-based approach: refers to the representation of a spatial domain by a discrete object rather than a continuous region. Typically, space is represented by a graph, that is, a set of ‘nodes’ connected pairwise by some ‘edges’, where nodes represent a salient object in the domain (e.g., a cell) and edges represent the fact of being spatial close (e.g., being the closest cell).
